# Patient and provider perspectives on barriers and facilitators to reproductive healthcare access for women experiencing homelessness with substance use disorders in San Francisco

**DOI:** 10.1177/17455057231152374

**Published:** 2023-02-22

**Authors:** Christina N Schmidt, Erin E Wingo, Sara J Newmann, Deborah E Borne, Brad J Shapiro, Dominika L Seidman

**Affiliations:** 1 School of Medicine, University of California San Francisco, San Francisco, CA, USA; 2 Department of Obstetrics, Gynecology & Reproductive Services, University of California San Francisco and San Francisco General Hospital, San Francisco, CA, USA; 3 San Francisco Department of Public Health, San Francisco, CA, USA; 4 Department of Psychiatry, University of California San Francisco, San Francisco, CA, USA

**Keywords:** family planning, homelessness, pregnancy, reproductive healthcare, substance use

## Abstract

**Background:**

Women experiencing homelessness with substance use disorders face unique and intersecting barriers to realizing their reproductive goals.

**Objective:**

This study explored the reproductive aspirations of this population, as well as the barriers to accessing reproductive services from the perspectives of affected individuals, and the healthcare providers who serve them.

**Design:**

This mixed-methods study included surveys and interviews with women experiencing homelessness with substance use disorders and healthcare providers.

**Methods:**

We conducted surveys and semi-structured interviews with women recruited from opiate treatment programs and homeless encampments in San Francisco, California in 2018. We also conducted interviews and focus groups with healthcare providers in reproductive health and substance use treatment settings. Interviews were recorded, transcribed, and coded. Descriptive statistics of survey results were performed.

**Results:**

Twenty-eight women completed surveys, 96% of whom reported current substance use. Ten women participated in interviews. One-third (9/28) reported desiring pregnancy in the next year; over half (16/28) reported they would be somewhat or very happy to learn they were pregnant. A majority used no contraception at last intercourse (14/28). Twenty-six healthcare providers participated in interviews (n = 15) and focus groups (n = 2). Patients and providers identified similar barriers to care access, including discrimination, logistical and financial challenges, and delayed pregnancy awareness. While providers proposed solutions focused on overcoming logistical challenges, patients emphasized the importance of transforming the healthcare environment to treat patients affected by substance use and homelessness with dignity and respect.

**Conclusion:**

Women experiencing homelessness with substance use disorders face intersecting and compounding barriers to accessing reproductive health services. For patients, the impact of stigma and bias on treatment experiences are particularly salient, in contrast to logistical barriers emphasized by providers. Improving access will require structural and individual-level solutions to address stigma and create person-centered, trauma-informed, and respectful care environments.

## Introduction

Women and girls comprise the fastest growing group of unstably housed individuals and account for almost 40% of the half a million people experiencing homelessness across the United States.^1^ Women experiencing homelessness face distinct and complex barriers to accessing reproductive health services, resulting in decreased use of preventive services—including contraception and prenatal care—compared to their housed peers.^2,3^ Traumatic reproductive healthcare experiences and hostile care environments disincentivize care engagement and contribute to medical distrust.^4,5^ Financial barriers and logistical challenges complicate access to clinic-based care.^3^ A long history of reproductive coercion in the United States directed toward marginalized communities, including the forced sterilization of minoritized populations, influences current dynamics around access to reproductive care.^6,7^ Beyond the numerous barriers to accessing services, women may need to prioritize basic survival including physical safety, shelter, and food over seeking reproductive health services.^3,8^ As the number of women experiencing homelessness increases, improving access to reproductive healthcare, including family planning and pregnancy care, for patients who desire these services is increasingly important.

High rates of substance use disorders (SUDs) further complicate access to reproductive care for women experiencing homelessness.^9,10^ In California, the state with the highest number of unsheltered people in the United States, 3 out of 4 unsheltered people report substance use.^11^ Among women with SUDs, judgment and stigma surrounding substance use disincentivizes access to reproductive services, and negative care experiences are common.^12–16^ For women experiencing homelessness who are pregnant, fear of losing child custody and criminalization of substance use in pregnancy contribute to care avoidance.^12,17–19^ Mental illness commonly co-occurs with homelessness and SUDs,^20^ and managing mental health symptoms may take precedence over patients’ reproductive health. Diverse types of past and ongoing trauma similarly affect this population, influencing engagement in care.^21^ Although trauma-informed practice has been increasingly prioritized in clinical settings, re-traumatization in healthcare is common and creates a powerful deterrent to care engagement due to well-founded fear of mistreatment and discrimination.^22^

Despite the frequent co-occurrence of homelessness, SUDs, and mental illness, at a systems level, supportive services are often siloed. Affected individuals frequently must present to multiple agencies or clinical sites with inconsistent accessibility and commitment to trauma-informed practice. This often results in patients having to complete duplicative intake processes and inconsistent or even conflicting care plans, as communication between supportive services is limited.

When describing the disparities in access to reproductive services among women experiencing homelessness and with SUDs, researchers often focus on higher rates of unintended pregnancies and lower rates of contraceptive use.^23,24^ While some individuals affected by homelessness and substance use may be interested in preventing pregnancy, overreliance on unintended pregnancy as a proxy for healthcare access obscures the complexity of each person’s family-building goals.^25^ Given the stigma and discrimination surrounding pregnancy and parenting among people experiencing homelessness, and especially those with SUDs, individuals may be less likely to report pregnancy intentions.^3^ Pregnancy intentions may also shift and change with time, and overreliance on singular, binary variables may lose the more nuanced understanding of reproductive goals.^26^ Understanding the reproductive aspirations of this population is an important step toward creating accessible, patient-centered services, and ensuring that the care offered matches patients’ reproductive goals.^27^ While studies exploring affected individuals’ reproductive goals are necessary to provide a nuanced assessment of patients’ healthcare needs, existing literature is limited.

Improving access to reproductive services requires partnership between patients and providers to identify barriers and propose solutions. While various studies have identified barriers to care from the perspective of affected individuals, few incorporate the perspectives of direct service providers, which is critical for redesigning care environments to meet the needs of patients.^28^ Consequently, we conducted this mixed-methods study to (1) explore the reproductive aspirations of women affected by homelessness and substance use and (2) explore both patients’ and providers’ perceived barriers to women’s accessing reproductive health services. By incorporating provider and patient perspectives, this study compares patient and provider priorities, with the goal of facilitating future interventions to improve access to high-quality, person-centered reproductive care.

## Methods

### Study design

This study was designed as a needs assessment in preparation for an intervention to improve reproductive health services of women affected by homelessness and substance use. We conducted surveys and semi-structured interviews with women in San Francisco affected by homelessness and substance use (patient participants) and interviews and focus groups with healthcare providers serving this patient population in reproductive health and substance use treatment settings (provider participants).

### Setting and participants

Patient participants were recruited from opiate treatment programs and homeless encampments in San Francisco, California, between December 2017 and January 2018 using convenience sampling. Study staff made announcements about the study at encampment health fairs or in clinic waiting rooms, and participants approached research staff to participate. Participants were included if they identified as experiencing homelessness (defined as staying on the street, tent, or vehicle; couch surfing—staying with friends; staying in a shelter; or staying in a residential treatment program). Verbal informed consent was obtained, and participants were excluded from the study if they were unable to consent to participate. Verbal consent was obtained rather than written consent so as not to assume that patients were literate. Participants were asked to repeat back key parts of the assent document to confirm their understanding. Consent was recorded and tracked in a secured and encrypted excel spreadsheet.

All eligible participants were invited to complete a survey regarding their reproductive aspirations, and participants who consented to complete a survey were sequentially invited to participate in a semi-structured interview. Interviews were presented as an opportunity to provide more details on reproductive health service experiences, in order to in future inform an intervention to improve these services. While transgender individuals were not excluded from this study, given the small sample size and the unique needs of this population, transgender individuals were not actively recruited as a subgroup participant population.

San Francisco-based frontline staff working in homeless health services, substance use treatment, and reproductive health settings were recruited to participate in either semi-structured interviews or focus groups between November 2017 and January 2018. Given the high prevalence of substance use among women experiencing homelessness in San Francisco, recruitment sites for provider participants included opiate treatment programs in addition to reproductive health and homeless health service settings. Provider participants were identified using snowball sampling, as well as general recruitment at staff meetings and via staff listservs, until a diversity of perspectives were achieved and participants’ responses reached saturation, reinforcing prior findings rather than introducing new themes.^29^

### Quantitative: reproductive aspirations survey

Patient participants who agreed to participate in the study completed a brief survey to assess their reproductive aspirations. Survey questions were developed and reviewed by co-authors with experience in the areas of obstetrics, family planning, homelessness, and addiction care. Participants provided demographic information (age, race), as well as length of residence in San Francisco, length of time spent homeless, and the location in which they currently slept. Participants reported current substance use and enrollment in substance use treatment programs. Participants also described their reproductive goals, past/present contraceptive use, and contraceptive preferences. Finally, the survey also explored how participants preferred to access reproductive health services (Supplement 1). Recruitment goals were based off of expert opinions by study investigators to support the needs assessment. In 2017, there were approximately 1000 unsheltered, reproductive-age cisgender women living in San Francisco, and the project aimed to recruit 3–5% of this population to complete surveys.^30^

### Qualitative: interviews

Interview questions were designed by co-authors based on their experiences providing care in obstetrics, family planning, homelessness, and addiction. Interview guides for patient participants included questions on contraception, abortion care, pregnancy, prenatal care, and childbirth. The interviewer had extensive experience working with people experiencing homelessness, as she had worked as an outreach worker for many years. Patient participants were asked to describe their past experiences using reproductive health services, discuss current barriers to accessing services, and offer suggestions for improving current care systems. Provider participants were asked to describe barriers to accessing reproductive health services for their patients and suggest solutions to address these barriers. All provider participants were also asked to provide basic demographic information including age, race/ethnicity, professional degrees, and any formal training in substance use or reproductive health. For patient participants, all interviews were conducted individually. For provider participants, data collection included interviews with individual service providers, as well as two focus groups with providers working in substance use treatment centers to facilitate increased participation and the diversity of respondents. All interviews and focus groups were audio recorded and transcribed.

### Statistical analyses

Survey results were analyzed using descriptive statistics. Interviews and focus groups were analyzed using a combination of deductive and inductive coding, using a thematic analysis approach.^31^ Initially, broad inductive codes were applied to the data (CS). A codebook was then created by CS and mutually reviewed and agreed upon by two members of the study team who reviewed the transcripts in depth (EW and CS). This codebook was then applied to all the transcripts by a single coder to ensure consistent application of the codes (CS). Codes were then examined to look for themes, and themes were reviewed and revised (CS, DS) to develop preliminary findings. A community advisory board (CAB) was founded following the completion of data collection in anticipation of intervention development, and the content of key themes was cross-referenced with detailed notes from the CAB’s discussions to assess for consistency of findings. All study team members reviewed the results prior to finalizing the analysis. Descriptive statistics were generated using R version 3.6.1. Qualitative data were analyzed using Atlas.ti version 8. The Consolidated Criteria for Reporting Qualitative Studies (COREQ) checklist for this study is included in Supplement 2.

### Ethics approval

Ethics approval was granted by the institutional review board of the University of California San Francisco (#17-23949). Patient participants who completed the survey were provided with a $5 gift card, and those who completed an interview were provided with a $10 gift card.

## Results

### Survey responses

Twenty-eight patient participants completed the reproductive aspirations and family planning preferences survey. The median age of participants was 30 years old. The majority (71%) of participants had experienced homelessness for over a year, and most (71%) reported spending some of this time living outside. All participants had a history of recent substance use, and 96% reported ongoing use at the time of the study, with 61% reporting heroin use, 46% reporting methamphetamine use, and 25% reporting cocaine use. One-third of participants were currently using methadone or buprenorphine for opioid use disorder (Table 1).

**Table 1.** Characteristics of survey participants.

Characteristic	n = 28^a^
Age	30 (27, 35)
Race	
Black	4 (14%)
Latina	1 (3.6%)
White	19 (68%)
Native American	1 (3.6%)
Pacific Islander	1 (3.6%)
Mixed	2 (7.2%)
Time spent experiencing homelessness	
<6 months	2 (7.2%)
6 months–1 year	5 (18%)
>1–5 years	11 (39%)
6–10 years	5 (18%)
>10 years	4 (14%)
Living situation	
Outdoors (sidewalk, tent, box)	20 (71%)
Vehicle (Car, RV)	3 (11%)
Shelter	6 (21%)
Single room occupancy	1 (3.6%)
Family or friend’s couch	4 (14%)
Rehabilitation center	1 (3.6%)
Current substance use	
Alcohol	6 (21%)
Marijuana	20 (71%)
Methamphetamines	13 (46%)
Heroin	17 (61%)
Fentanyl	5 (18%)
Benzodiazepines	6 (21%)
Hallucinogens	2 (7.2%)
Other opioids^b^	3 (11%)
Cocaine	7 (25%)
Medication for opioid use disorder	
Methadone	7 (29%)
Buprenorphine^c^	2 (7.2%)
None	19 (68%)

a Statistics presented: Median (IQR); n (%).

b Other opioids includes oxycodone, hydrocodone, morphine, and so on.

c The survey did not differentiate between taking buprenorphine versus buprenorphine-naloxone.

One-third of patient participants desired pregnancy in the next year (32%) and half (50%) did not desire pregnancy. Conversely, over half (57%) reported that they would be very or somewhat happy if they discovered that they were pregnant at the time of survey completion. Half of participants were using contraception. Of those who reported using a pregnancy-prevention strategy, half were using withdrawal as their primary approach. The majority of respondents (82%) were either very or somewhat happy with their current method of contraception. If all contraceptive methods were easily accessible and available, the most desired method of contraception was birth control pills (29%). In addition, 29% of participants reported that they would not be interested in contraception even if they had access to all methods of contraception.

Over a third (36%) of patient participants reported that they would prefer to receive contraception at a clinic or hospital specialized in women’s health, although women also selected clinics they attend for other care (21%), places they go for social services (21%), or a provider visiting the place where they stay (18%) as their preferred location for accessing contraception (Table 2).

**Table 2.** Reproductive aspirations and contraceptive preferences, survey results.

Characteristic	n = 28^a^
Desire pregnancy in the next year	
Yes	9 (32%)
No	14 (50%)
Unsure	5 (18%)
Feeling if discovered pregnancy today	
Very happy	9 (32%)
Somewhat happy	7 (25%)
Somewhat upset	1 (3.6%)
Very upset	7 (25%)
Unsure	4 (14%)
Current method of pregnancy prevention^b^	
Condoms	3 (11%)
Withdrawal	7 (25%)
Birth control pills	0 (0.0%)
Injectables	1 (3.6%)
Intrauterine device	2 (7.2%)
Implant	0 (0.0%)
Vaginal ring	0 (0.0%)
Morning after pill	0 (0.0%)
Vasectomy	1 (3.6%)
Fertility awareness method	0 (0.0%)
None	14 (50%)
Happiness with current method	
Very happy	5 (18%)
Somewhat happy	18 (64%)
Somewhat unhappy	5 (18%)
Very unhappy	0 (0.0%)
Desired contraception if available	
Condoms	6 (21%)
Birth control pills	8 (29%)
Injectables	4 (14%)
Intrauterine device	3 (11%)
Implant	2 (7.2%)
Tubal ligation	1 (3.6%)
Fertility awareness method	4 (14%)
None	8 (29%)
Best place to access contraception	
Having a nurse/doctor come to the place I stay	5 (18%)
At a women’s health clinic or hospital	11 (36%)
At a clinic I go to for other care	6 (21%)
At a place I go to for other social services	6 (21%)

a Statistics presented: n (%).

b Participants were asked to select all methods used.

### Interviews and focus groups

Of the 28 patient participants who completed surveys, 10 agreed to participate in an in-depth interview conducted at encampments (n = 6) and opiate treatment programs (n = 4). Six had been pregnant in the past, two had experienced a miscarriage, and two had prior abortions. Nine of the 10 patient participants were not using a medical birth control method.

Twenty-six healthcare providers participated in interviews (n = 15) and two focus group discussions (focus groups included 11 additional providers). Provider participants worked in women’s health clinics (n = 4), opiate treatment programs (n = 12), and street medicine or homeless services (n = 10). Interviewees included counselors/outreach workers (n = 11), doctors/nurse practitioners (n = 8), nurses (n = 4), and medical directors/administrators (n = 3). Half (50%) of the providers had formal training in substance use treatment, 35% had formal training in family planning, and 27% had formal training in homelessness. Both patient and provider participants discussed perceived barriers to accessing reproductive services, as well as potential solutions to improve access to care (Table 3).

**Table 3.** Characteristics of providers focus groups and interview participants.

Characteristics	Focus group participants, n = 11^a^	Interview participants, n = 15^a^	Total, n = 26^a^
Work location			
Women’s health clinic	0 (0.0%)	4 (27%)	4 (15%)
Opiate treatment program	11 (100%)	1 (6.7%)	12 (46%)
Street medicine or homeless services	0 (0.0%)	10 (67%)	10 (38%)
Role			
Counselor or outreach worker	8 (73%)	3 (20%)	11 (42%)
Doctor or nurse practitioner	3 (27%)	5 (33%)	8 (23%)
Nurse	0 (0.0%)	4 (27%)	4 (15%)
Medical director or administrator	0 (0.0%)	3 (20%)	3 (12%)
Professional training^b^			
Family planning	0 (0.0%)	9 (60%)	9 (35%)
Homelessness	0 (0.0%)	7 (47%)	7 (27%)
Substance use	11 (100%)	2 (13%)	13 (50%)

a Statistics presented: n (%).

a Participants could select training in more than one area.

#### Barriers to accessing reproductive health services: patient and provider perspectives

Patient and providers respondents generally described similar barriers to accessing contraception and reproductive health services. The themes that were described by all participants—both patients and providers—included (1) judgment and discrimination, (2) logistical and financial challenges, and (3) delayed pregnancy awareness.

1. Judgment and discrimination**—**Both patient and provider participants described judgment and discrimination as primary factors disincentivizing care-seeking. Patients universally described feeling disrespected in past encounters with reproductive healthcare providers and/or substance use treatment settings. When describing her experiences working with outreach service teams, one respondent expressed her frustration with staff members and providers that she frequently interacted with, stating:

Even in the programs, the people that work there, it’s like why are you here if you are just here to torment us?

When describing her most recent birth, another participant commented on the lack of respect that she felt during her hospital stay, saying:

We are going through enough as it is, who knows about our situation? We know it’s tough, we are not proud of it that said, I really think [providers] in this field who deal with people like me should be trained . . . They are the ones who call for harm really, they are the ones that put things into our heads, and they did a lot of damage.

Providers similarly described disrespect as primary barriers to accessing reproductive health services. Providers described judgment and stigma arising from participants “looking homeless.” One provider described the profiling of women experiencing homelessness at a women’s health clinic in the city, and noted that patients are often treated differently based on their appearance:

I’ll just say it very boldly—it’s not a clinic where a disheveled looking person or a young-looking person or a confused or distressed looking person can go in there and be sure that someone will at least think carefully about whether they can do something for them today.

Another provider reflected on the impact this profiling and judgment on the experiences of patients in clinical settings, stating:

Some is just the social part of it that our patients look homeless, and for them to try to interact with the medical industry in general they feel like they don’t belong there, and people don’t want to take care of them. They don’t like how they’re treated.

Providers also described the judgment arising from patients’ substance use and how past negative experiences in healthcare settings and experiences of stigma disincentive future care-seeking. One provider described the impacts of a positive urine drug test on the way that patients are treated in clinical environments:

A big barrier on clinic side is that clients have had past experiences of being judged by providers. They feel stigma as a drug user and don’t want to engage in care somewhere that they might be judged. One positive urine [toxicology screening] and the staff’s demeanor changes. The way staff treats them at some places, it makes them feel so bad that they would rather have sex without protection than put themselves through that judgment and shaming of going to the clinic.

Patient participants described similar feelings of judgment from reproductive health providers because of their substance use. When describing her interactions with healthcare staff, one patient participant mentioned the response of staff on a labor and delivery floor to her use of methadone during her pregnancy:

People need to be educated about it because there’s a lot of judgment around it and it makes me, honestly, kind of want to start getting off of it. But I know that that would be detrimental to me.

2. Logistical and financial challenges**—**Experiencing logistical and financial barriers to accessing care was another prominent theme arising from both patient and provider participants. Patients mainly focused on challenges related to paying for services, resulting from lack of money or difficulty obtaining insurance coverage. One patient described the financial cost associated with starting birth control, stating:

They wanted like three hundred and ten dollars. I was like, I can’t even afford it, I can’t afford three [dollars]. Crazy.

Providers similarly stressed that financial resources and getting patients signed up for insurance coverage is particularly challenging for women who are transiently housed. When describing the barriers patients face to accessing care, one provider stated:

A lot of it’s financial, a lot of them are so transient that they don’t have [Medicaid]. They don’t have any kind of insurance coverage. The logistics of access are tough.

Providers also highlighted that the location and hours of services are not currently set up in a way that facilitates access to care for people experiencing homelessness and SUDs. The challenges described matched those reported by patients completing the quantitative surveys; barriers discussed included difficulties with transportation, as well as limited clinic hours. As one provider working at a women’s health clinic stated:

We run our clinic by appointment. We have open and closed hours. That doesn’t fit with folks’ needs.

3. Delayed pregnancy awareness**—**Both providers and patients described delays in pregnancy discovery as a common barrier to accessing prenatal care. Participants explained that women are often not aware that they are pregnant until the late second or third trimester. Patient participants described denial of their pregnancies, a theme that was reaffirmed by providers. Multiple women stated that they did not want to believe that they could be pregnant, due to fear. One woman described her most recent pregnancy:

Yeah. I mean I didn’t really, I should have known . . . I kept saying it that it wasn’t really . . . you know I hadn’t known how to take care of it and everything, and I was too scared to take a test you know? And I didn’t really have a person to talk to because I was staying with my grandma. I didn’t even have a choice in that.

Providers similarly described denial as a driver of delayed pregnancy awareness. One provider expanded on this point:

I think there are a lot of internal barriers again for a woman who would like to keep the pregnancy and would like to have a life where she could keep the pregnancy. I’ve seen a number of patients that don’t allow themselves to realize they’re pregnant until they’re pretty darn pregnant.

Multiple providers also attributed delayed awareness to participants being disconnected from their bodies, due to trauma or active substance use. One provider described a recent patient who presented to their opiate treatment program:

She didn’t realize until about seven months that she was pregnant, and she gave birth like three and a half weeks later . . . But she didn’t feel like it—it wasn’t—she didn’t think about it as a possibility . . . I think a lot of our patients don’t have a relationship with their body and their self-awareness of what’s going on with their body. It’s definitely been affected by years of drug use and trauma so they’re not really necessarily in touch with what’s going on with them physically.

#### Facilitators to accessing reproductive health: patient and provider perspectives

Both provider and patient participants emphasized that care coordination and high-touch case management facilitate care engagement. One patient participant described her close relationship with her social worker during her most recent pregnancy:

I found my friend in the [outreach] team. She became my friend really. She directed me everywhere. They go with me to [the hospital], they got me in with them. Yeah, they basically trained me in all directions . . . they made a lot of care for me. I think the whole team is a great team I mean it’s not a word you would think of during pregnancy.

Providers similarly described how intensive case management teams and accompaniment to appointments facilitates attendance at reproductive health visits and promotes better clinical experiences. A counselor at one of the opiate treatment programs commented on the value to accompaniment at reproductive care visits:

Have a staff member or peer navigator available to walk clients over to their abortion or prenatal visits and sit with them. Anecdotally, counselors have done this and found it successful . . . Having advocates who go with patients to their appointments . . . the doctor is more respectful with someone else in the room who is a professional.

#### Proposals to improve access to care: provider perspectives

When discussing possible solutions to improve access to reproductive services, providers generally focused on solutions aimed at overcoming logistical challenges. This included co-locating services, improving service hours, providing drop-in services, facilitating transportation, strengthening case management programming, and increasing dedicated staff resources. Multiple providers described shifting to open-access clinic models, which would allow participants to receive care without scheduling an appointment. One provider described:

I’m a huge fan of the open access model. I just want that regular clinic schedule for people to be able to walk into. It doesn’t change the provider’s burden of patients; it just changes how people access it. I’ve had a lot of success in our Street Medicine open access clinic.

Many providers also suggested bringing services to the locations that clients are already visiting. A provider working at an opiate treatment program suggested that reproductive services be provided to clients at their clinic:

Clients are already here every day seeing excellent medical providers, we are missing an opportunity to provide them with birth control.

Providers also mentioned the importance of bolstering case-management services to improve attendance rates at clinic visits. One provider working at a women’s health clinic described the value of having social workers at clinics to offer services:

It would be great at [our clinic] if we had social workers that were dedicated to housing needs or psychosocial needs. Our counselors can’t work with people on an ongoing basis. But this might be a time in a patient’s life where it’s her only interaction with health care if she’s actively using and on the streets. It would be so great if we could offer more in-depth services.

#### Proposals to improve access to care: patient perspectives

While providers focused on logistical changes to improve access to care, patient participants predominantly emphasized the importance of transforming the healthcare environment and creating spaces where patients are treated with dignity and respect. One participant described the importance of demonstrating compassion toward patients, rather than creating hostile environments:

Ninety-five percent of them would work with [patients affected by homelessness or substance use] as if they are working against them. Because it is just that they are highly combative. Show them that you actually care . . . you need some doctors that do know how to deal with that. Even if they come here stinking, don’t turn them away, just help them.

Patient respondents suggested interventions aimed at improving provider training with the goal of creating respectful and trauma-informed care environments. One participant described the importance of training healthcare workers to be sensitive to the unique challenges resulting from living on the street:

[It is important for staff] to be more educated about harm reduction and the trauma that being homeless can bring, and how that can affect your mood and to be compassionate to those issues.

Overall, patient participants emphasized the importance of changing the healthcare environment so that patients affected by homelessness and substance use are treated with the same respect as all other patients seeking reproductive health services. When referencing the improvements that healthcare providers can make to better serve this patient population, one participant stated:

All of their messages . . . could we just be treated like any other patients? You know [we] are normal people just like you and anybody else.

## Discussion

This study explored the reproductive aspirations of women affected by homelessness and substance use, as well as the barriers to accessing reproductive services, from the perspectives of affected individuals and the healthcare providers who serve them. Structured as a needs assessment, our goal was to inform future interventions seeking to improve reproductive healthcare for this growing patient population. Our quantitative results revealed that the reproductive aspirations of affected individuals are diverse, with almost a third of patient participants in our sample reporting that they would desire pregnancy in the next year, and half stating they would be happy if they found out they were pregnant today. Half of patient participants were not using contraception, and the majority (82%) were at least somewhat happy with their current contraceptive use. Our qualitative findings identified similar barriers to accessing reproductive services from patients and providers—including discrimination, logistical challenges, and delayed pregnancy awareness—yet proposed diverging solutions to improve access to care.

Our quantitative findings about women’s reproductive aspirations, and specifically how more respondents anticipated feeling satisfied with finding out they were pregnant today than those who reported desiring a future pregnancy, are consistent with the evolving literature around evaluation of pregnancy intentions. In a study by Ralph et al., investigators used the London Measure of Unplanned Pregnancy—a validated tool for assessing pregnancy intention—to compare retrospective and prospective measurements of pregnancy intention. The investigators found that retrospectively women were less likely to describe their pregnancies as unintended and that the circumstances of participants’ lives influenced their likelihood of describing a pregnancy as unintended.^26^ Furthermore, while a pregnancy might not be intended, it may still be a positive outcome for patients.^32^ Our study emphasizes the importance of exploring reproductive goals and aspirations in various ways, particularly with patient populations who experience significant stigma and discrimination around their reproduction, to best understand and support individual’s reproductive intentions.

Moreover, while our study initially focused on patients’ access to family planning, our findings indicated broader interest in improving access to reproductive services in general, including trauma-informed preconception and pregnancy care. Uptake of contraception and prevention of unintended pregnancies are frequently used as metrics to quantify access to reproductive healthcare.^33^ However, placing a disproportionate emphasis on these metrics obscures the heterogenous reproductive aspirations of this population. Increased access to reproductive health services will not result in uptake of a new contraceptive method if patients are satisfied with their current care, do not desire contraception, or desire pregnancy. Research that focuses narrowly on access to family planning will not reflect, and may even obscure, the complexity of patients’ reproductive aspirations. For patients who do desire pregnancy, early engagement in care may be vital to supporting parenting goals, given the numerous challenges associated with supporting a newborn while experiencing homelessness and managing SUDs. Ultimately, when re-envisioning how to offer reproductive health services, the diversity of patients’ reproductive aspirations and pregnancy desires must be considered.

In the qualitative portion of our study, patients and providers identified similar barriers to accessing reproductive health services. Both providers and patients identified three primary drivers of poor access to adequate reproductive healthcare: (1) discrimination and stigma, (2) logistical and financial challenges, and (3) delayed pregnancy awareness. While other studies have revealed similar barriers to those identified in our sample,^3,4,28^ our study was unique in evaluating barriers to accessing reproductive health services from the perspective of both affected individuals and their direct service providers.

While patients and providers identified similar barriers to accessing reproductive care, the solutions proposed by the two cohorts differed significantly. Providers proposed solutions focused on overcoming logistical challenges, with ideas ranging from drop-in appointments, to transportation services, to dedicated case managers and social workers. For patients who desire access to contraception, for example, outreach vans and co-location of services may increase access to care. Contrastingly, patients emphasized the importance of transforming the healthcare environment to treat individuals affected by homelessness and substance use with dignity and respect. Solutions envisioned by patients included improving the training of providers and staff, with the goal of educating providers on the realities of homelessness and substance use and decreasing judgment and discrimination in healthcare settings.

The discordance in solutions proposed by participants with lived experience reflects one of the primary challenges in increasing access to healthcare services for underserved populations. Focusing exclusively on logistical changes does not address one of the primary causes of decreased participation in care from the perspective of patients—discrimination and negative experiences with the healthcare system. While interventions aimed at increasing the accessibility of services through logistical changes may decrease some of the existing barriers to care, addressing logistical and financial barriers in isolation is insufficient. Logistical interventions must be coupled with efforts to reshape the culture of systems of care.

Care that center the voices, autonomy, and lived experiences of patients is an important step toward building more respectful and compassionate care experiences for affected populations. Trauma-informed practice offers principles to guide such efforts. While definitions vary, underlying concepts include recognizing the high prevalence of traumatic experiences (including traumatic healthcare experiences) among patients, providers, and staff, and fostering a care environment that is safe, transparent and trustworthy; that supports collaboration and choice; and that is responsive to and supportive of those with intersecting identities.^34–36^ Healing-centered engagement, coined by Dr. Shawn Ginwright, takes those concepts one step further. Healing centered engagement is an asset-driven approach, specifically recognizing that affected individuals’ identities are more than their past traumas, and acknowledging and addressing underlying structural causes of collective and community trauma.^37^

Trauma-informed practice and healing-centered engagement approaches may include interventions to address the logistical barriers to care as proposed by provider participants, while concurrently promoting responsive services that offer options and choice. In addition, these approaches focus explicitly on the care environment as proposed by patient participants, to ensure a safe, trustworthy, and respectful experience. Thus, principles of trauma-informed practice may bridge some of the discordant findings proposed by patient and provider participants in this project, and critically emphasize centering the perspectives of those with lived experience.

### Limitations

Study limitations include a small sample size in one city. With this sample size, the views of study participants cannot be presumed to reflect the greater population of women experiencing homelessness with SUDs in San Francisco. Our use of snowball sampling is an additional limitation, as this form of participant recruitment may result in a clustering of experiences from members within the same community and may limit the diversity of responses. Moreover, due to our method of recruitment through presentations at staff meetings or encampment health fairs, we were unable to track the number of people who declined to participate, which would allow better understanding of who was missing from our study. Additional research across a larger population and including strategies to track those who decline to participate may help to validate our study’s findings and increase generalizability.

In addition, our sample was limited to patients and providers recruited in an urban center facing intersecting housing and substance use crises. California hosts over half of the United States’ homeless population, and rates of homelessness in the state are continuing to rise.^1^ While the high incidence of homelessness and substance use made San Francisco an ideal site for this investigation, San Francisco also invests heavily in public health, and access to services and commitment to supporting the reproductive health and well-being of this patient population may differ across locations. The results of this study must be considered within the context of our study location.

Our study only included cisgender women, and additional research examining the experiences of transgender individuals are necessary to understand the needs of this unique population. Furthermost, although this study addressed multiple of the intersecting factors that contribute to reproductive health service access among women experiencing homelessness and using substances, additional factors such as engagement in transactional sex, mental illness, and history of trauma likely influence care experiences, and future studies could explore how these factors uniquely contribute.

## Conclusion

Dominant narratives surrounding access to reproductive health services assume that women affected by homelessness and substance use do not desire pregnancy, and that lack of contraceptive uptake is driven by barriers to accessing reproductive services. While affected individuals experience numerous barriers to accessing reproductive care, our study reveals that the reproductive aspirations of women are diverse, and that many desire pregnancy. Both patients and providers in this study acknowledged similar intersecting and compounding barriers to accessing reproductive services. However, the solutions that they proposed to increase care accessibility differed significantly. Providers primarily focused on logistical solutions, while patients emphasized the importance of transforming the care environment. The results of this study suggest that solutions focused exclusively on eliminating logistical barriers to accessing reproductive health services are insufficient. Without investment in changing provider attitudes and fostering healing care environments, improved access to person-centered reproductive health services for women affected by homelessness and substance use will not be fully realized.

## Supplemental Material

sj-docx-1-whe-10.1177_17455057231152374 – Supplemental material for Patient and provider perspectives on barriers and facilitators to reproductive healthcare access for women experiencing homelessness with substance use disorders in San Francisco Click here for additional data file. Supplemental material, sj-docx-1-whe-10.1177_17455057231152374 for Patient and provider perspectives on barriers and facilitators to reproductive healthcare access for women experiencing homelessness with substance use disorders in San Francisco by Christina N Schmidt, Erin E Wingo, Sara J Newmann, Deborah E Borne, Brad J Shapiro and Dominika L Seidman in Women’s Health

sj-docx-2-whe-10.1177_17455057231152374 – Supplemental material for Patient and provider perspectives on barriers and facilitators to reproductive healthcare access for women experiencing homelessness with substance use disorders in San Francisco Click here for additional data file. Supplemental material, sj-docx-2-whe-10.1177_17455057231152374 for Patient and provider perspectives on barriers and facilitators to reproductive healthcare access for women experiencing homelessness with substance use disorders in San Francisco by Christina N Schmidt, Erin E Wingo, Sara J Newmann, Deborah E Borne, Brad J Shapiro and Dominika L Seidman in Women’s Health

## Declarations

**Ethics approval and consent to participate:** Ethics approval was granted by the institutional review board of the University of California San Francisco (#17-23949). Verbal informed consent to participate was obtained from all participants.

**Consent for publication:** Not applicable.

**Availability of data and materials:** Data supporting the findings of this study are available from the corresponding author DLS on request. The data are not publicly available due to identifiable information that could compromise the privacy of research participants.
