# Supplementary material for: Patient and provider perspectives on barriers and facilitators to reproductive healthcare access for women experiencing homelessness with substance use disorders in San Francisco
Source: Womens Health (Lond). 2023 Feb 22;19:17455057231152374. doi: 10.1177/17455057231152374 (PMC9947686; doi:10.1177/17455057231152374)
Supplement: sj-docx-1-whe-10.1177_17455057231152374 – Supplemental material for Patient and provider perspectives on barriers and facilitators to reproductive healthcare access for women experiencing homelessness with substance use disorders in San Francisco [file sj-docx-1-whe-10.1177_17455057231152374.docx]

**Reproductive aspirations survey**

1. How old are you? ____________
2. What race do you identify with most? Check all that apply:
   1. White
   2. Black
   3. Latina
   4. Asian
   5. Pacific Islander
   6. Native American
   7. Mixed
   8. Other/Prefer not to answer
3. How long have you been in San Francisco?
4. Less than one month
5. 1-6 months
6. 6 months to one year
7. More than one year: ___________________ *(write in # yrs)*
8. How long have you been homeless?
   1. Less than one month
   2. 1-6 months
   3. 6 months to one year
   4. More than one year: ___________________ *(write in # yrs)*
9. Where do you sleep?
   1. Outdoors: sidewalk, tent or box
   2. Car, truck, or RV
   3. A shelter or navigation center
   4. A friend/family member’s couch
   5. SRO room
   6. Other: ____________
10. What methods of birth control have you used in the past? (Check all that apply)
    1. Male condoms
    2. Pulling out
    3. Female condoms
    4. Birth control pill
    5. Morning after pill/Plan B
    6. Depo shot
    7. Patch
    8. Vaginal ring/nuvaring
    9. IUD
    10. Nexplanon/rod in your arm
    11. None of the above
    12. Other:__________________
11. The last time you needed to see a doctor for birth control, pregnancy care, or abortion care, where did you go?

*[write in response]*

1. How was that experience?
   - 1. I liked it and would go there again
     2. I didn’t like it that much but would still go there again
     3. I didn’t like it and would not go back
        1. Why? *[write in response]*
2. Where would be the best place for you to get you birth control or pregnancy care? *[Circle top choice]*
   1. Having a nurse or doctor come to the place where I stay
   2. At a clinic or hospital that specializes in women’s health
      1. *Which one?*
   3. At a clinic that I go to for other care, like methadone clinic
      1. *Which one?*
   4. At a place I go to for other services, like Homeless Prenatal or Ladies Night, Needle Exchange,
      1. *Which one?*
   5. Other idea? _________________________
3. Do you prefer to drop-in for birth control or pregnancy care, or have a scheduled appointment?
   1. Drop in
   2. Scheduled in advance
4. What is the best way for you to schedule an appointment?
   1. By calling the clinic
   2. By texting
   3. By dropping in and making an appointment
   4. By a website
   5. By email
   6. Other: ___________________
5. If there was one thing we could do to make it easier for you to get birth control or pregnancy care, what would it be?

*Write in:*

1. Circle any of the drugs below that you have used in the last month
2. Alcohol
3. Marijuana
4. Crystal meth
5. Heroin
6. Fentanyl
7. Oxycodone, Percocet, Tylenol #3, Morphine, or other opiate bought on the street
8. Xanax or other benzo
9. Crack cocaine
10. Powder cocaine
11. Ketamine
12. GHB
13. Molly
14. Hallucinogens such as mushrooms/Acid/LSD
15. None
16. Other:__________________
17. Are you in a methadone program?
    1. YES *where? ______________________*
    2. NO
18. Are you prescribed buprenorphine from a healthcare provider?
    1. YES
    2. NO
19. Are you pregnant right now?
    1. YES 🡪 please answer the questions in the box below
    2. **NO 🡪** please skip the questions in the box and move to **question #29**

If you are **pregnant** right now, please answer these questions:

1. How far along were you when you found out you were pregnant?
2. Did you wish you had found out sooner?
   1. Yes
   2. No
3. Where did you find out you were pregnant?
   1. In private with an at home pregnancy test
   2. In a clinic visit
   3. At another place where I go to get services
4. Were you trying to get pregnant?
   1. Yes
   2. No
5. Have you given birth before?
   1. Yes 🡪 how long ago?
   2. No
6. How did you feel when you found out about this pregnancy?
   1. Very happy
   2. Somewhat happy
   3. Somewhat upset
   4. Very upset
   5. I don’t know
7. Have you gotten checked out for your pregnancy?
   1. Yes
   2. No
8. How will you get checked out for your pregnancy, if at all?
   1. I don’t want prenatal care
   2. I’d go to a place I drop in for services and ask them what to do (for example, Larkin St., Resource Center, etc)
   3. I’d talk to the HOT team when they come by.
   4. I’d go to Homeless Prenatal
   5. I’d go to the methadone clinic
   6. I’d walk into a clinic *[write in which one]*
   7. I’d call to make an appointment at a clinic *[write in which one]*
   8. I’d go to the emergency room *[write in which one]*
   9. Other: __________________________
9. If you wanted to get an abortion, what would you do?
   1. I don’t want an abortion
   2. I’d go to a place I drop in for services and ask them what to do (for example, Larkin St., Resource Center, etc) *[write in which one]*
   3. I’d talk to the HOT team when they come by.
   4. I’d go to the methadone clinic
   5. I’d walk into a clinic *[write in which one]*
   6. I’d call to make an appointment at a clinic *[write in which one]*
   7. I’d try to do something myself *[write in what]*
   8. Other: __________________________
10. If you wanted to give your baby up for adoption, what would you do?
    1. I don’t want to do adoption
    2. I’d go to a place I drop in for services and ask them what to do (for example, Larkin St., Resource Center, etc) *[write in which one]*
    3. I’d talk to the HOT team when they come by.
    4. I’d go to the methadone clinic
    5. I’d walk into a clinic *[write in which one]*
    6. Other: __________________________
11. After pregnancy, do you think you’ll be interested in starting a birth control method?
    1. Yes
    2. No
12. IF YES, which of the following, if any, would you be interested in using?
    1. Male condoms
    2. Female condoms
    3. Birth control pill
    4. Morning after pill/Plan B
    5. Depo shot
    6. Patch
    7. Vaginal ring/nuvaring
    8. IUD
    9. Nexplanon/rod in your arm
    10. Getting my tubes tied
    11. Fertility awareness method
    12. None of the above
    13. Other:__________________

If you are **not pregnant** right now, continue here:

1. Would you like to become pregnant in the next year?
   1. YES
   2. NO
   3. UNSURE
2. If you found out you were pregnant today, how would you feel?
   1. Very happy
   2. Somewhat happy
   3. Somewhat upset
   4. Very upset
   5. I don’t know
3. If you found out you were pregnant today and wanted to keep the baby, would you want to go get checked out for your pregnancy?
   1. YES
   2. NO
4. IF YES, how would you do that?
   1. I’d go to a place I drop in for services and ask them what to do (for example, Larkin St., Resource Center, etc)
   2. I’d talk to the HOT team when they come by.
   3. I’d go to Homeless Prenatal
   4. I’d go to the methadone clinic
   5. I’d walk into a health clinic *[write in which one]*
   6. I’d call to make an appointment at a clinic *[write in which one]*
   7. I’d go to the emergency room *[write in which one]*
   8. Other: __________________________
5. If you found out you were pregnant today and wanted to get an abortion, what would you do?
   1. I’d go to a place I drop in for services and ask them what to do (for example, Larkin St., Resource Center, etc)
   2. I’d talk to the HOT team when they come by.
   3. I’d go to the methadone clinic
   4. I’d walk into a health clinic *[write in which one]*
   5. I’d call to make an appointment at a clinic *[write in which one]*
   6. I’d try to do something myself *[write in what]*
   7. Other: __________________________
6. Thinking about the last time you had sex, did you do anything to try to avoid getting pregnant?
   1. No
   2. I had anal or oral instead of vaginal sex
   3. My partner pulled out
   4. My partner used a condom
   5. I used the morning after pill/Plan B afterwards
   6. I’m using birth control (circle which one)
      1. Birth control pills
      2. Patch
      3. Vaginal ring/Nuvaring
      4. IUD
      5. Implanon/Nexplanon/rod in my arm
      6. Female condom
      7. Depo shot
   7. Other:__________________________
7. [***If answered NO to #34 above***] If you didn’t do anything to avoid getting pregnant, why not?
8. I want to get pregnant
9. My partner didn’t want to use anything
10. I didn’t want to use anything
11. I didn’t have anything with me at the time
12. Other: ________________________
13. ***[Referring to the method used in #9, even if it was no method]*** How happy are you with your current method of preventing pregnancy?
    1. Very happy
    2. Somewhat happy
    3. Somewhat unhappy
    4. Very unhappy
14. If the following methods of birth control were **available to you to start using today**, which of the following, if any, would you be interested in using?
    1. Male condoms
    2. Female condoms
    3. Birth control pill
    4. Morning after pill/Plan B
    5. Depo shot
    6. Patch
    7. Vaginal ring/nuvaring
    8. IUD
    9. Nexplanon/rod in your arm
    10. Getting my tubes tied
    11. Fertility awareness method
    12. None of the above
    13. Other:__________________
15. Where would you go if you decided you wanted to start using a new birth control method that you needed a doctor or nurse to start using?
    1. I’d go to a place I drop in for services and ask them what to do (for example, Larkin St., Resource Center, needle exchange, etc). *[write in which one]*
    2. I’d talk to the HOT team when they come by.
    3. I’d go to the methadone clinic and ask them
    4. I’d walk into a clinic *[write in which one}*
    5. I’d call to make an appointment at a clinic *[write in which one]*
16. Other: __________________________

****That is the last question. Thank you for your time!****

**Would you like a handout with info about:**

*** Birth control options?**

*** Women’s health clinics?**

*** Pregnancy options including abortion, adoption, and prenatal care?**

**🡪 If yes, ask the person who gave you this survey!**
