# Supplementary material for: Patient and provider perspectives on barriers and facilitators to reproductive healthcare access for women experiencing homelessness with substance use disorders in San Francisco
Source: Womens Health (Lond). 2023 Feb 22;19:17455057231152374. doi: 10.1177/17455057231152374 (PMC9947686; doi:10.1177/17455057231152374)
Supplement: sj-docx-2-whe-10.1177_17455057231152374 – Supplemental material for Patient and provider perspectives on barriers and facilitators to reproductive healthcare access for women experiencing homelessness with substance use disorders in San Francisco [file sj-docx-2-whe-10.1177_17455057231152374.docx]

| **Consolidated criteria for reporting qualitative research (COREQ) Checklist** | |
| --- | --- |
| 1. Interviewer/facilitator | Nora Anderson |
| 2. Credentials | At the time of the interviews the interviewer has an MPA in health policy management. |
| 3. Occupation | At the time of the interviews the interviewer worked at UCSF as a research program manager. |
| 4. Gender | The interviewer was Female. |
| 5. Experience and training relationship with participants | The interviewer had 3 years of experience working as a research project manager, including in the Departments of Family Medicine and Obstetrics and Gynecology, where she managed multiple large research projects and conducted qualitative interviews. She also had 3 years of experience as a patient navigator for people experiencing homelessness with substance use disorders. She also has experience as a doula. |
| 6. Relationship established | The interviewer had no relationship with participants prior to beginning the research study. |
| 7. Participant knowledge of the interviewer | The participants were verbally consented by the interviewer. They were told she was interested in learning about the reproductive care experiences of women experiencing homelessness, with the goal of improving access to care. |
| 8. Interviewer characteristics | When introducing herself to participants, the interviewer stated that she was a researcher and had previously worked in street outreach. Although she did not explicitly describe herself as a woman, participants may have deduced this information about her when the met or talked with her. Her interest in participating in this project came from her prior experience working at the intersection of street-based medicine and reproductive health services. |
| 9. Methodological orientation and theory | This study used thematic analysis. |
| 10. Sampling | To recruit patient-participants, staff made announcements about the study at encampment health fairs or in clinic waiting rooms, and participants were invited to approach research staff to participate. Participants were included if they identified as experiencing homelessness (defined as staying on the street, tent, or vehicle; couch surfing – staying with friends; staying in a shelter; or staying in a residential treatment program).  Provider participants were identified using snowball sampling until a diversity of perspectives were achieved and participants’ responses reached saturation. |
| 11. Method of approach | Patient-participants were approached in-person at opiate treatment programs and reproductive health and homeless service centers.  Provider-participants were recruited at staff meetings and via staff listservs (email). |
| 12 Sample Size | Twenty-eight patient-participants participated in the study (10 completed interviews in addition to surveys).  Twenty-six provider-participants participated in the study. |
| 13. Non-participation | Our recruitment strategy for patients was opt-in; once participants approached the team to opt-in, all were enrolled in and completed the study. All participants who were invited to participate completed the study. |
| 14. Setting of data collection | All interviews were conducted in person. Patient interviews were conducted at opiate treatment centers and encampments, based on patient preference. Provider interviews were also conducted in person. |
| 15. Presence of non-participants | During most patient interviews the interviewer was the only person present. However, because some of these interviews took place at homeless encampments, some interviews took in semi-private locations, as requested by participants (i.e. a location where other family members were present).  Provider interviews were conducted a participants’ respective workplaces. |
| 16. Description of sample data collection | Twenty-eight patient-participants experiencing homelessness and using substances participated in this study.  Twenty-six providers working in women’s health clinics, opiate treatment programs and street medicine or homeless services participated. Interviewees included counselors/outreach workers, doctors/nurse practitioners, nurses and medical directors/administrators.  Participants completed interviews between December 2017 and January 2018. |
| 17. Interview guide | Semi-structured interviews were conducted based on an interview guide. The interview guide was reviewed by a diverse group of experts in reproductive health, addiction, homeless health and street medicine prior to the start of the study. |
| 18. Repeat interviews | No repeat interviews were conducted. |
| 19. Audio/visual recording | Audio recording was used to record interviews. |
| 20. Field notes | Field notes were made during and after interviews. |
| 21. Duration | Interviews lasted approximately 20-30 minutes. |
| 22. Data Saturation | We reached data saturation when we could not identify any additional codes. |
| 23. Transcripts returned | Transcripts were not returned to participants. However, we reviewed all findings with a community advisory board, comprised of patients with lived experience. |
| 24. Number of data coders | One coder coded all data. |
| 25. Description of the coding tree | The initial codebook was developed by a single research based on broad inductive coding of the data, and then reviewed and agreed upon by multiple study team members (see 26). |
| 26. Derivation of themes | Interviews and focus groups were analyzed using a combination of deductive and inductive coding, using a thematic analysis approach. Initially, broad inductive codes were applied to the data. A codebook was then created by and mutually reviewed and agreed upon by two members of the study team who reviewed the transcripts in depth. This codebook was then applied to all the transcripts by a single coder to ensure consistent application of the codes. Codes were then examined to look for themes, and themes were reviewed and revised to develop preliminary findings. |
| 27. Software | We used Atlus.ti for our qualitative analysis. |
| 28. Participant checking reporting | Participants did not directly provide feedback on the findings; however we reviewed all findings with our Community Advisory Board, comprised of patients with lived experience. |
| 29. Quotations presented | Quotations were presented with a description of participant role/occupation, to illustrate themes and key findings while keeping data anonymized. |
| 30.Data and findings consistent | We summarize key findings and include quotations, to highlight major and minor themes. |
| 31. Clarity of major themes | Major themes are described in the paper. |
| 32. Clarity of minor themes | There is a description of diverse cases within relevant themes. |
